# Electrocatalytic C–N Coupling for Urea Synthesis

**DOI:** 10.1002/smsc.202100070

**Published:** 2021-10-15

**Authors:** Chen Chen, Nihan He, Shuangyin Wang

**Affiliations:** ^1^ State Key Laboratory of Chem/Bio-Sensing and Chemometrics College of Chemistry and Chemical Engineering Hunan University Changsha P. R. China

**Keywords:** C—N bonds, coupling reactions, electrocatalysis, molecule fixations, urea syntheses

## Abstract

The industrial urea synthesis consists of two consecutive processes, nitrogen + hydrogen → ammonia followed by ammonia + carbon dioxide → urea. The electrocatalytic coupling of carbon source (carbon dioxide) and nitrogen source (nitrogen, nitrite, nitrate) by skipping the ammonia synthetic process might be a promising alternative to achieve the efficient urea synthesis; in this case, two industrial steps with high energy consumption and high pollution are optimized into one renewable energy‐driving electrocatalytic process. Herein, the progress of green urea synthesis is summarized, focusing on the electrocatalytic coupling of carbon source and nitrogen source for direct urea synthesis under ambient conditions. The mechanism researches for urea synthesis are also reviewed, and the future development directions of electrocatalytic urea synthesis are prospected. The electrocatalytic C–N coupling reaction realizes the efficient resource utilization and provides guidance and reference for molecular coupling reactions.

## Graphical abstract

The industrial urea production requires two‐step complex synthetic processes with high energy consumption and carbon dioxide emission. The electrocatalytic C–N coupling strategy greatly simplifies the synthesis method of urea, serving as the promising alternative for urea synthesis. To realize the high efficiency and high yield rate of urea production, much effort needs to be put.

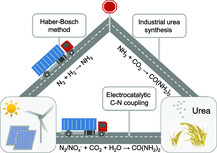

## 1 Introduction

The industrial synthesis of chemicals usually operates under harsh conditions with high energy consumption, aggravating energy crisis and environmental concerns.^[^ ^1^ ^]^ Driven by renewable electricity, the electrocatalysis can reduce the energy barrier of the reaction to achieve the efficient and green synthesis of chemicals under milder conditions.^[^ ^2^ ^]^ The realization of the resource utilization of abundant gaseous molecules through electrocatalytic activation and fixation is the focus of electrocatalysis research nowadays.^[^ ^3^ ^]^ Among them, the electrocatalytic conversion of carbon dioxide can achieve the electrosynthesis of liquid fuels with high energy density, which not only alleviates the energy shortage, but also contributes to progress toward the carbon‐neutral goal.^[3b]^ As the key intermediate for electrocatalytic carbon dioxide reduction, the direct electrolysis of carbon monoxide could be an efficient alternative approach to achieve the synthesis of multicarbon products with promoted carbon–carbon coupling.^[^ ^4^ ^]^ However, the electrolysis of carbon dioxide or carbon monoxide only yields products with limited bond structure, whereas the synthesis of valuable chemicals containing special bonds (such as C—N bond) still suffers from the drawbacks of rigorous reaction conditions.

**Figure 1:**
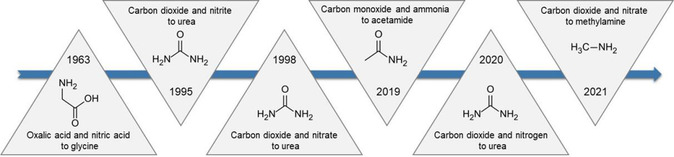
The progress of C–N coupling reactions for electrocatalytic amine synthesis.

To this end, Jiao and collaborators creatively introduced ammonia as another reactant into the electrocatalytic reduction of carbon monoxide (**Figure** **1**). The formation of C—N bond arises from the nucleophilic attack of ammonia to the *C═C═O intermediate, resulting in the production of acetamide with nearly 40% efficiency.^[^ ^5^ ^]^ This strategy of combining electrocatalytic processes with organic reactions has been adopted to broaden the application of C—N bond coupling, which can be dated back to the year of 1963. Rapson and Bird demonstrated the electrosynthesis of glycine from the electroreduction of oxalic acid and nitric acid in the same cell.^[^ ^6^ ^]^ The interaction between the electrochemically generated glyoxylic acid and hydroxylamine realizes the C–N coupling and desirable yield of glycine. By expanding the substrates, Fukushima and Yamauchi achieved the efficient synthesis of a series of amino acids via conducting the electrolysis of α‐keto acids with ammonia/hydroxylamine.^[^ ^7^ ^]^ As an important building block for valuable chemicals, methylamine is always produced from high temperature and pressure reaction of methanol and ammonia. Very recently, Wang et al. achieved the direct electrosynthesis of methylamine from coelectrolysis of carbon dioxide and nitrate via the condensation of hydroxylamine from nitrate reduction and formaldehyde from carbon dioxide reduction,^[^ ^8^ ^]^ as shown in **Figure** **1**.

**Figure 2:**
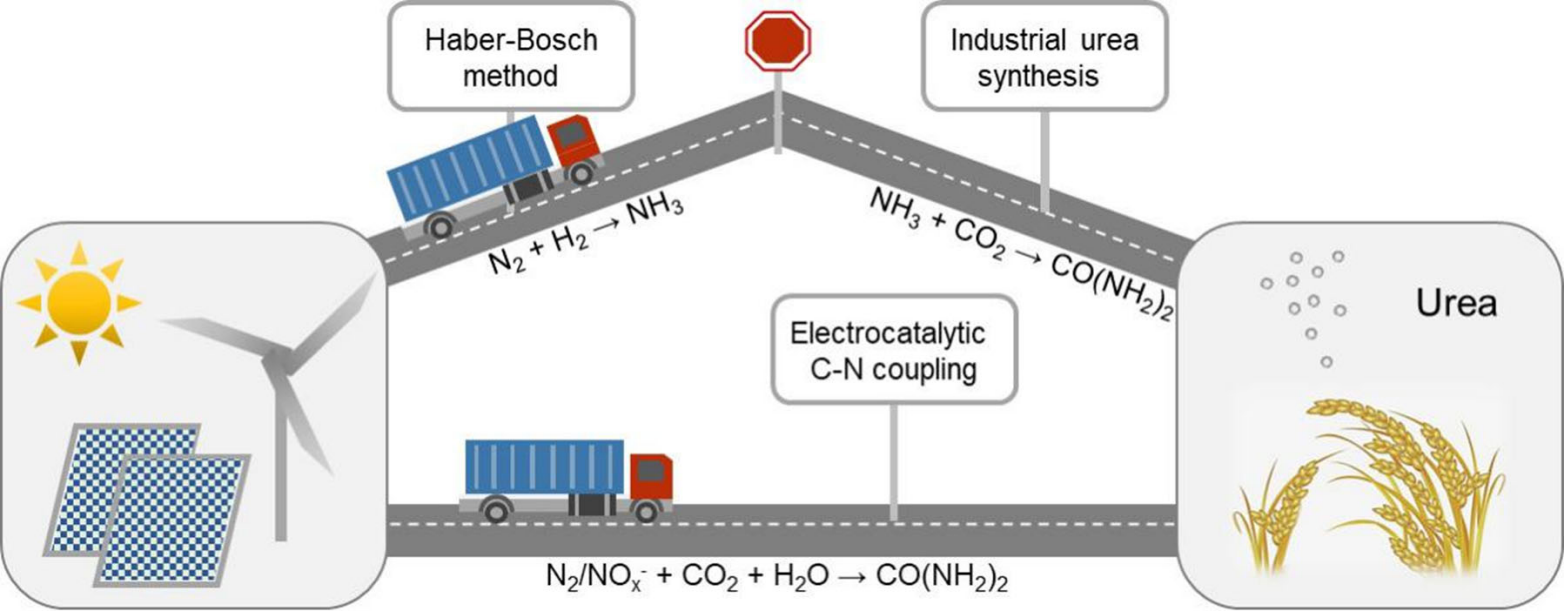
Pathways to urea synthesis. Haber–Bosch method combined with industrial urea synthesis (scenario A) and direct electrocatalytic C–N coupling process (scenario B).

It is a feasible and effective strategy to realize the C—N bond formation and amines generation by coelectrolysis of carbon‐containing and nitrogen‐containing feedstocks.^[^ ^5^, ^6^, ^7^, ^8^ ^]^ As one of the important amines in the fields of agriculture and industry, the conventional synthesis of urea includes two consecutive processes.^[^ ^9^ ^]^ Nitrogen reacts with hydrogen to form ammonia and ammonia reacts with carbon dioxide to form urea, both operating at high temperature and high pressure (scenario A in **Figure** **2**). The ammonia synthesis through Haber–Bosch method consumes about 2% of the global energy annually; in addition, the feedstock of hydrogen is obtained from reforming of the fossil fuels, belching out hundreds of millions of tons of carbon dioxide annually.^[^ ^1^ ^]^ The pursuit for the ammonia synthesis under milder conditions did not stop over the past century.^[^ ^10^ ^]^ The electrocatalytic process was demonstrated as the promising alternative for its renewable driven force and the protons can be gained from water splitting rather than gaseous hydrogen.^[^ ^2^, ^3^ ^]^ However, the electrocatalytic ammonia synthesis is still in its initial stage with low yield due to high chemical inertness of nitrogen.^[^ ^2^ ^]^ Urea is the main downstream product of ammonia, the separation and purification of ammonia from aqueous media for subsequent urea synthesis face tremendous challenges as well.

Therefore, the exploration of the electrocatalytic process for the direct synthesis of urea is needed. In this review, we summarize the progress of green urea synthesis, focusing on the electrocatalytic coupling of carbon source (carbon dioxide) and nitrogen source (nitrogen, nitrite, nitrate) for direct urea synthesis under ambient conditions (scenario B in Figure 2). The mechanism researches for urea synthesis are also reviewed, and the future development directions of electrocatalytic urea synthesis are prospected.

## 2 Coupling of Carbon Dioxide and Nitrogen for Urea Synthesis

**Figure 3:**
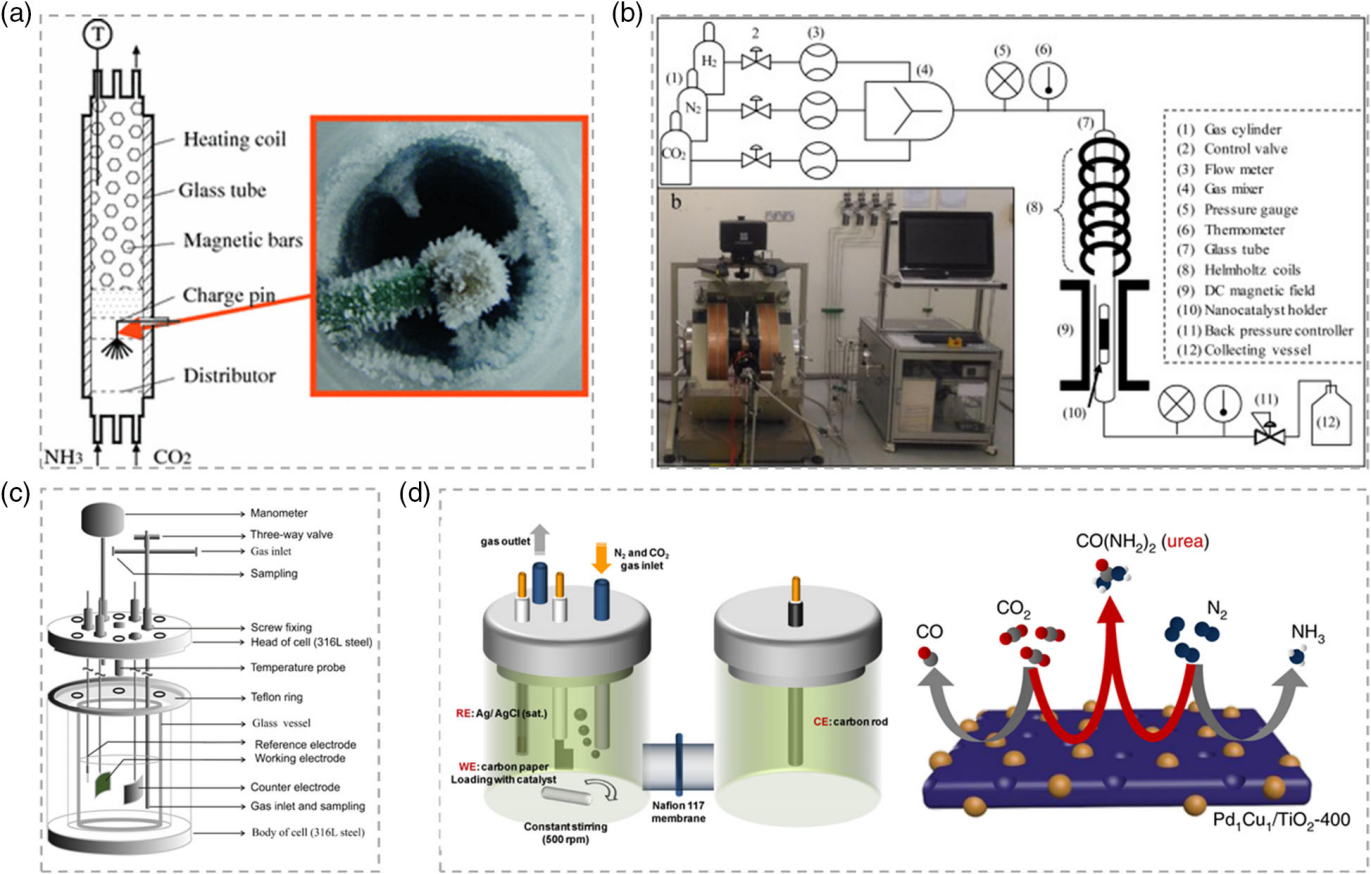
Strategies toward efficient urea synthesis. a) Plasma‐assisted urea synthesis from carbon dioxide and ammonia under atmospheric pressure. Reproduced with permission.^[^ ^13^ ^]^ Copyright 2012, Springer Nature. b) Electromagnetic field‐induced urea production from carbon dioxide, nitrogen, and hydrogen at ambient conditions. Reproduced with permission.^[^ ^14^ ^]^ Copyright 2020, Elsevier Ltd. c) Electrocatalytic synthesis of urea from carbon dioxide and nitrogen in a pressurized reactor. Reproduced with permission.^[^ ^15^ ^]^ Copyright 2016, Elsevier Ltd. d) Electrocatalytic coupling of carbon dioxide and nitrogen to synthesize urea under ambient conditions. Reproduced with permission.^[^ ^12^ ^]^ Copyright 2020, Springer Nature.

The gaseous nitrogen is the abundant source of nitrogen, which makes up 78% of the atmosphere. Due to high inertness of nitrogen, the utilization of nitrogen is dominated by the Haber–Bosch method to synthesize ammonia at high temperature and high pressure (400–500 °C, 200–300 bar).^[^ ^11^ ^]^ As the main downstream product of ammonia, urea is formed by the reaction of ammonia and carbon dioxide along with C—N bond formation, which is also conducted at harsh conditions (150–200 °C, 150–250 bar).^[^ ^12^ ^]^ Considering the high equipment requirements and complex synthetic processes of the industrial method, strategies for more efficient urea synthesis have to be explored. Xiang et al. achieved urea synthesis at atmospheric pressure by the plasma‐assisted method.^[^ ^13^ ^]^ The production of urea arises from the reaction between electronegative ammonia anions and carbon dioxide, and the solid product of urea could be obtained in the reactor, as shown in **Figure** **3**a. However, the plasma‐assisted synthesis of urea is difficult to overcome the problem of energy consumption for driving the plasma generator,^[^ ^2^ ^]^ and the green manufacture of ammonia has not yet been realized.

The “one‐step” conversion of carbon dioxide and nitrogen by skipping the ammonia synthetic process might be a promising alternative to achieve efficient urea synthesis. Alqasem et al. demonstrated the electromagnetic field‐induced one‐step urea synthesis at ambient conditions but it still suffers from the drawback of using hydrogen as the feedstock.^[^ ^14^ ^]^ Utilizing aqueous solution instead of hydrogen as the proton source would exploit the advantages of green urea synthesis and it was realized in a pressurized apparatus, as shown in Figure 3c.^[^ ^15^ ^]^ In the mixed atmosphere of carbon dioxide (30 bar) and nitrogen (30 bar), the urea formation rate reaches the highest value of 31.8 μg h^−1^ cm^−2^ over the polypyrrole catalyst at −0.325 V (vs. normal hydrogen electrode (NHE)). Shifting the high‐pressure working condition to an ambient one would minimize the requirements for synthesis equipment as much as possible. Thus, the green synthesis method of carbon dioxide and nitrogen coupling to produce urea under ambient temperature and atmospheric pressure arises at the right moment. Chen et al. demonstrated electrocatalytic urea synthesis on a Pd_1_Cu_1_/TiO_2_‐400 catalyst with a urea formation rate of 0.12 mmol g^−1^ h^−1^ and corresponding Faradic efficiency of 0.66% at −0.4 V (vs. reversible hydrogen electrode (RHE)).^[^ ^12^ ^]^

**Figure 4:**
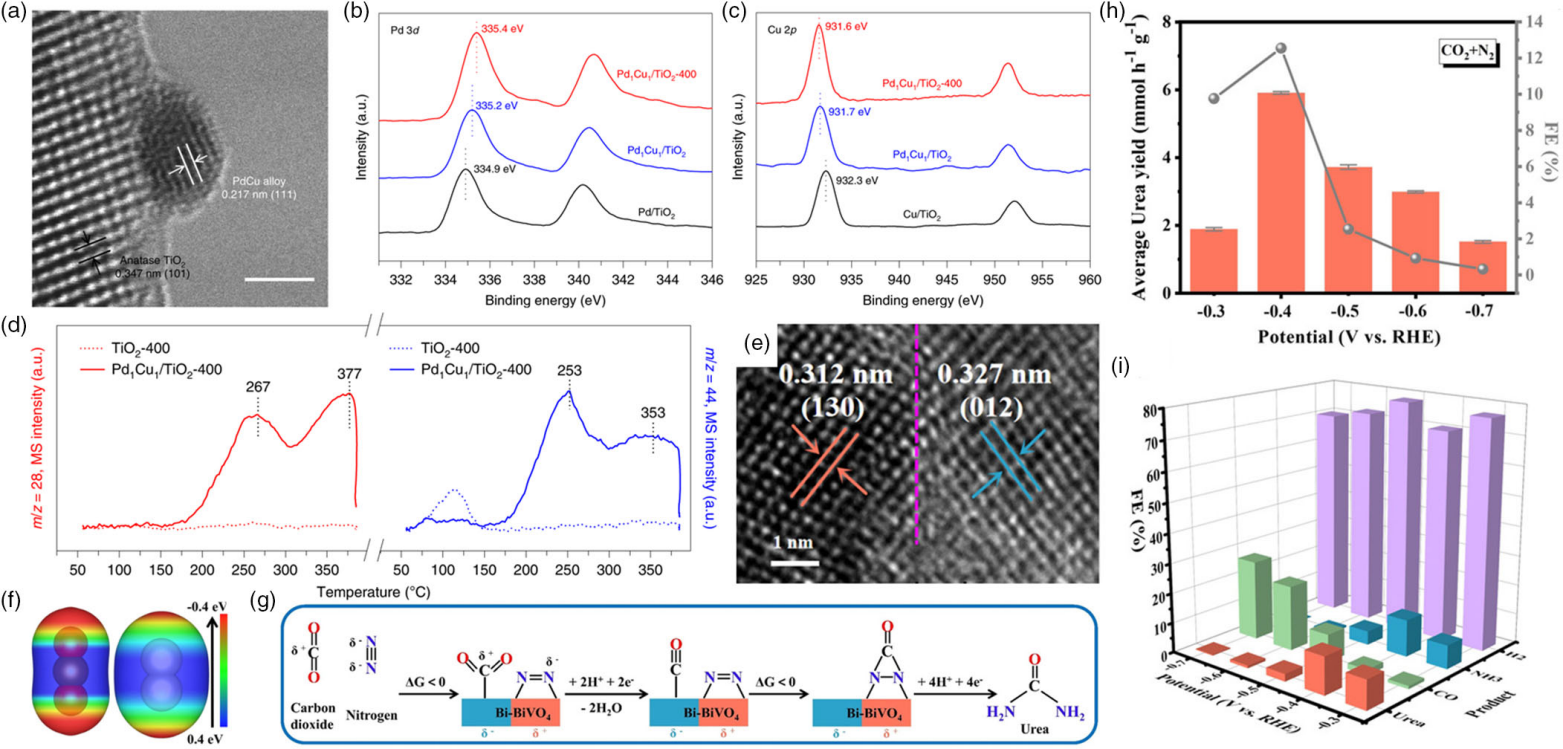
Design of electrocatalysts for coupling of nitrogen and carbon dioxide for urea synthesis. a) High‐resolution TEM image of Pd_1_Cu_1_/TiO_2_‐400 catalyst. b) Pd 3*d* XPS spectra of Pd/TiO_2_, Pd_1_Cu_1_/TiO_2_, and Pd_1_Cu_1_/TiO_2_‐400. c) Cu 2*p* XPS spectra of Cu/TiO_2_, Pd_1_Cu_1_/TiO_2_, and Pd_1_Cu_1_/TiO_2_‐400. d) Competitive chemisorption of nitrogen and carbon dioxide on TiO_2_‐400 and Pd_1_Cu_1_/TiO_2_‐400. a–d) Reproduced with permission.^[^ ^12^ ^]^ Copyright 2020, Springer Nature. e) High‐resolution TEM image of Bi/BiVO_4_ hybrid. f) Electron density isosurface of carbon dioxide and nitrogen molecules. g) The proposed reaction pathway of urea formation on Bi/BiVO_4_ hybrid. h) The urea yield rate and i) the corresponding product distribution at various potentials for Bi/BiVO_4_ hybrid. e–i) Reproduced with permission.^[^ ^18^ ^]^ Copyright 2021, Wiley‐VCH.

To enhance the electrocatalytic synthesis rate of urea, the design of electrocatalyst and the optimization of catalytic system are needed. Metal alloying can promote the electronic interactions between components and provide more active sites for electrocatalytic reaction,^[^ ^16^ ^]^ and the electronic interactions would be further enhanced by loading the metal alloy on the defect‐rich support,^[^ ^12^, ^17^ ^]^ as confirmed by the shifting of binding energy in **Figure** **4**b,c. The authors demonstrated that the coadsorption of nitrogen and carbon dioxide can be realized on the catalyst, which is the prerequisite for urea formation.^[^ ^12^ ^]^ The strategy of catalyst design for urea synthesis should focus on the coadsorption and activation of gaseous molecules simultaneously. Yuan et al. provided a strategy for coactivation and urea synthesis by heterostructure constructing,^[^ ^18^ ^]^ as shown in Figure 4e. The electron‐rich N atom in nitrogen and electron‐deficient C atom in carbon dioxide would preferentially adsorb on the electrophilic and nucleophilic regions in Bi/BiVO_4_ hybrids, attributed to the electronic interactions between the reactive molecules and hybrid structure (Figure 4f,g). The urea yield rate of 5.91 mmol g^−1^ h^−1^ and Faradic efficiency of 12.55% are obtained over this catalyst at −0.4 V (vs. RHE). Due to the accelerated local charge redistribution and enhanced molecular adsorption capacity in heterostructure, Yuan et al. also demonstrated the perovskite BiFeO_3_/BiVO_4_ hybrids as another efficient electrocatalyst for urea electrochemical synthesis under ambient conditions.^[^ ^19^ ^]^

**Figure 5:**
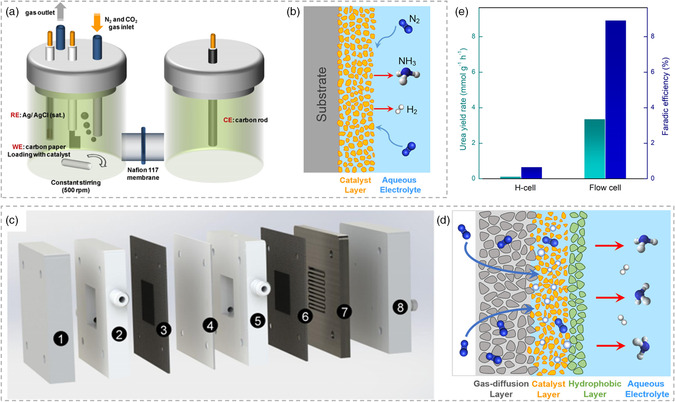
The optimization of catalytic system for urea synthesis. a) The typical H cell for urea electrocatalytic synthesis. Reproduced with permission.^[^ ^12^ ^]^ Copyright 2020, Springer Nature. b) Schematic illustration of solid–liquid interface in H cell. Reproduced with permission.^[^ ^21^ ^]^ Copyright 2020, American Chemical Society. c) Electrochemical flow cell for urea synthesis. Reproduced with permission.^[^ ^12^ ^]^ Copyright 2020, Springer Nature. d) Proposed solid–liquid–gas interface in a flow cell with gas‐diffusion electrodes. Reproduced with permission.^[^ ^21^ ^]^ Copyright 2020, American Chemical Society. e) Comparison of electrocatalytic performance in a H cell and a flow cell.

Electrocatalytic urea synthesis is a gas consumption reaction and the yield rate of urea is limited by the low solubility of gas, especially nitrogen.^[^ ^20^ ^]^ Meanwhile, the surface of electrode is dominantly covered by water molecules, which is beneficial for the competitive hydrogen evolution reaction.^[^ ^21^ ^]^ In addition to the rational design of the electrocatalyst, the optimization of the catalytic system is equally important. **Figure** **5**a shows the typical H cell with continuous gas bubbling during the electrochemical measurements. The gaseous feedstocks need to dissolve in the aqueous solution and then diffuse to the electrode surface for participation in the reaction,^[^ ^21^ ^]^ as shown in Figure 5b. The electrochemical flow cell with gas‐diffusion layer could construct an efficient three‐phase reaction interface, allowing the gas and liquid from different channels to encounter the solid catalysts for reaction. Moreover, the hydrophobic layer in Figure 5d blocks most of the water molecules, only permitting a small number of them to pass through as a source of protons for urea synthesis.^[^ ^12^, ^21^ ^]^ Chen et al. tested the urea synthesis performance in an H cell and a flow cell, respectively. The results in Figure 5e show that both the yield rate of urea and the Faradic efficiency are obviously improved due to the abundant gaseous feedstocks and better suppressed competitive reaction, affording another pathway regarding catalytic system optimization for urea synthesis.^[^ ^12^ ^]^

**Figure 6:**
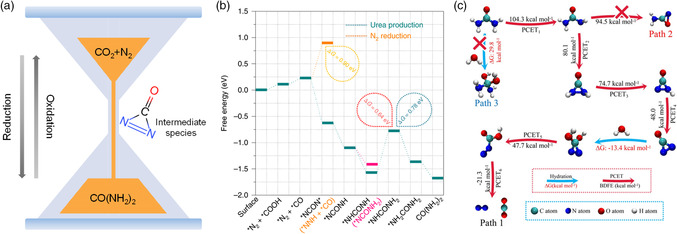
The proposed reaction mechanisms for electrocatalytic urea generation and degradation. a) The schematic diagram for electrocatalytic urea transformation. b) The proposed mechanism for urea synthesis from nitrogen and carbon dioxide reduction. Reproduced with permission.^[^ ^12^ ^]^ Copyright 2020, Springer Nature. c) The proposed mechanism for urea oxidation to nitrogen and carbon dioxide. Reproduced with permission.^[^ ^22^ ^]^ Copyright 2021, Wiley‐VCH.

The electrocatalytic urea production from nitrogen and carbon dioxide coupling involves multistep electrochemical processes and nonelectrochemical processes. Relevant studies are essential to clarify the reaction path and mechanism, so as to provide guidance for the design of electrocatalysts.^[^ ^12^, ^18^ ^]^ The electrochemical urea synthesis at elevated pressure was achieved by Kayan and collaborators.^[^ ^15^ ^]^ They proposed that urea production arises from the chemical reaction between carbon dioxide and ammonia from nitrogen reduction, and then the generated ammonium carbamate is converted to urea with one water molecule release. However, the driven force and the inherent mechanism for the generation of carbamate and urea have not been studied yet. Based on the results of theoretical calculations, Chen et al. demonstrated that the adsorbed nitrogen molecules would facilitate the carbon dioxide reduction process to form carbon monoxide and the migrated carbon monoxide species would react with side‐on adsorbed nitrogen.^[^ ^12^ ^]^ This reaction is exothermic and thermodynamically feasible and induces the formation of the key intermediate of *NCON* (**Figure** **6**a,b), serving as the precursor for urea generation. The competition reaction of *NNH formation is highly endothermic with an energy input of +0.90 eV, corresponding to the experimental results with greatly suppressed release of the side product ammonia. By means of isotope labeling operando electrochemical measurements, the evolution of bond structures in intermediate species has been tracked and the formation of key C—N bond has been confirmed experimentally. Yuan's work also displayed the feasibility of this reaction mechanism for urea synthesis on various electrocatalysts.^[^ ^18^, ^19^ ^]^

Urea can be obtained by electrocatalytic coupling of nitrogen and carbon dioxide, and the electroxidation of urea usually produces nitrogen and carbon dioxide as the main products. There is an internal relationship between urea synthesis and oxidation process. In terms of urea oxidation, Chen et al. proposed its reaction mechanism in Figure 6c based on isotope labeling experiments and theoretical results.^[^ ^22^ ^]^ Oxidative dehydrogenation occurs first in the amino groups of urea, followed by the intramolecular coupling of N—N bond. It should be noted that the key intermediate of *NCON* also forms before the fraction of C—N bond, thus resulting in the high proportion of nitrogen product. The electrochemical behaviors of urea production and oxidation are tightly related to the particularity of urea molecules with spatial relative positions of nitrogen atoms. According to the previous results of mechanism studies, the adsorption configuration of the nitrogen molecules, the reaction paths, and product types of carbon dioxide reduction and formation of the key intermediate (*NCON*) play vital roles for the understanding of inherent principles toward electrocatalytic urea synthesis from nitrogen and carbon dioxide coupling.

## 3 Coupling of Carbon Dioxide and Nitrite/Nitrate for Urea Synthesis

The production and living activities of human beings have a great impact on the Earth environment, among which, a large number of nitrogen‐containing substances are discharged into the surface water in the forms of nitrite and nitrate, threatening human health.^[^ ^23^ ^]^ The conventional approach involves catalytic conversion of nitrite and nitrate ions into nonpolluting nitrogen molecules.^[^ ^24^ ^]^ Although the harmless treatment of harmful substances has been achieved, however, it cannot realize the transformation and upgrading of materials and the maximum utilization of energy and waste resources. Subsequent researches are focused on the electrocatalytic reduction of nitrite and nitrate ions into ammonia, also meeting the problem for fabrication of the downstream product as mentioned earlier.^[^ ^25^ ^]^ Electrocatalytic coupling of carbon dioxide and nitrite/nitrate is an effective way to achieve the above purposes. The expansion of raw material to nitrate can not only realize the resource utilization of carbon dioxide, but also help to control water pollution, and finally realize the efficient green synthesis of urea.

The electrochemical reduction of carbon dioxide and nitrite for urea synthesis was first reported by Shibata et al. using Cu‐loaded gas‐diffusion electrode. With 1 bar carbon dioxide and 0.02 m nitrite ions as feedstocks, the Faradic efficiency of 37% has been achieved at the applied potential of −0.75 V (vs. standard hydrogen electrode (SHE)).^[^ ^26^ ^]^ Then, the reactivity of urea synthesis on zinc electrode was investigated, and the nitrite ion concentration, pressure of carbon dioxide, and the reaction temperature for urea production have been optimized.^[^ ^27^ ^]^ The authors found that cadmium electrode, zirconium boride, and Ni–phthalocyanine are beneficial for catalyzing urea synthesis from carbon dioxide and nitrite ions coreduction, and the Faradic efficiency for this reaction can reach the highest values of 55%, 33%, and 40%, respectively.^[^ ^28^ ^]^ With nitrate ions as the nitrogen source, electrochemical measurements on the gas‐diffusion electrodes with various catalysts were carried out and the maximum Faradic efficiency of urea formation on zinc catalysts is ≈35% at −1.75 V.^[^ ^29^ ^]^ Authors also investigated the electrochemical behavior of urea synthesis by carbon dioxide and nitrate‐ion coupling over metallophthalocyanine catalysts.^[^ ^30^ ^]^ The carbon monoxide formation rate from carbon dioxide reduction at Ni–phthalocyanine catalyst is significantly improved than that at metal catalyst. However, these catalysts have poor abilities to reduce nitrate ions to nitrite ions and ammonium ions, hindering the electrochemical urea synthesis. Thus, it was confirmed that simultaneous reduction of carbon dioxide and nitrate ions is necessary in this coupling reaction.^[^ ^30^ ^]^

**Figure 7:**
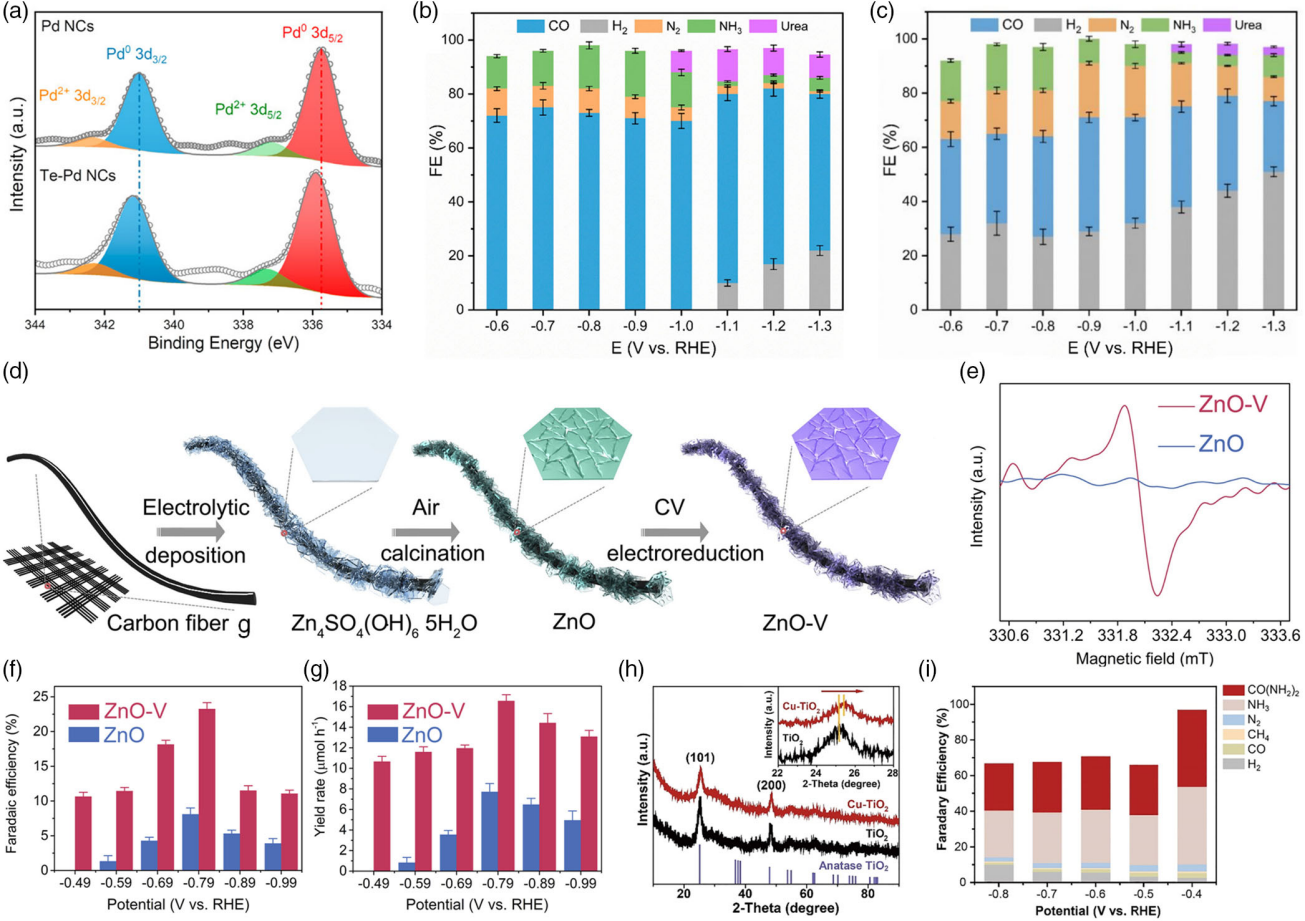
Recent progress of electrocatalytic urea production by coupling carbon dioxide and nitrite ions. a) Pd 3*d* XPS spectra of Te–Pd NCs and Pd NCs. Faradic efficiency versus the applied potentials for urea electrosynthesis on b) Te–Pd NCs and c) Pd NCs. a–c) Reproduced with permission.^[^ ^31^ ^]^ Copyright 2020, American Chemical Society. d) Schematic illustration of synthesis of ZnO–V porous nanosheets. e) EPR spectra of ZnO and ZnO–V porous nanosheets. Faradic efficiencies and urea yield rates under the given potentials over f) ZnO and g) ZnO–V. d–g) Reproduced with permission.^[^ ^32^ ^]^ Copyright 2021, Elsevier Ltd. h) XRD results for undoped TiO_2_ and Cu–TiO_2_–V_o_. i) Faradic efficiencies of the major reduction products for Cu–TiO_2_–V_o_ catalyst. h,i) Reproduced with permission.^[^ ^33^ ^]^ Copyright 2020, Elsevier Ltd.

Recently, new progress has been made in the production of urea by the coupling of carbon dioxide and nitrite ions. Feng et al. demonstrated Te‐doped Pd nanocrystals (NCs) for efficient electrochemical urea production.^[^ ^31^ ^]^ The 3*d*_3/2_ and 3*d*_5/2_ peaks of Pd^0^ exhibit a small negative shift for Te–Pd NCs, suggesting the electron transfer from Te to Pd (**Figure** **7**a). Due to the tailoring of the electronic structure, the carbon dioxide adsorption and the ammonia production were promoted by Te doping, inducing urea production with high efficiency.

The introduction of oxygen vacancy into the electrocatalyst is an efficient strategy to boost the electrochemical performance, resulting from the increase of active sites and the improved adsorption of feedstock and the enhanced stabilization of intermediate species.^[^ ^12^, ^32^, ^33^ ^]^ Meng et al. successfully prepared the oxygen vacancy‐rich ZnO porous nanosheets via the electroreduction of pristine ZnO precursor,^[^ ^32^ ^]^ as shown in Figure 7d. The electron spin resonance (EPR) signal at *g* = 1.95 corresponding to oxygen vacancies is observed in ZnO (Figure 7e). Compared with the ZnO nanosheets counterpart, the electrocatalytic performances of urea synthesis on ZnO–V nanosheets were obviously improved; among them, the Faradic efficiency of urea production can reach 23.26% at −0.79 V (vs. RHE). Heteroatom doping can also introduce abundant oxygen vacancies into the oxide electrocatalyst. Cao et al. demonstrated that the low‐valence Cu doped into TiO_2_ nanotubes could yield an oxygen vacancy‐rich catalyst.^[^ ^33^ ^]^ The X‐ray diffraction (XRD) peaks of Cu–TiO_2_–V_o_ gradually shift toward higher diffraction angles, suggesting the decrease in the TiO_2_ lattice constant, as shown in Figure 7h. The obviously increasing ratio of Ti^3+^/Ti^4+^ confirms the introduction of abundant oxygen vacancies, inducing efficient nitrite adsorption and activation on TiO_2_; meanwhile, the low‐valence Cu dopants served as effective catalytic sites to reduce carbon dioxide into active species. This oxygen vacancy‐rich Cu–TiO_2_ electrocatalyst enables high urea production rate (20.8 μmol h^−1^) and corresponding Faradic efficiency (43.1%) at a low potential of −0.4 V (vs. RHE). The performances of electrocatalytic coupling of carbon source (carbon dioxide) and nitrogen source (nitrogen, nitrite, nitrate) for direct urea synthesis are shown in **Table** **1**.

**Table 1:** The performances of electrocatalytic coupling of carbon source and nitrogen source for urea synthesis

Catalyst^[ref.]^	Carbon source	Nitrogen source	Electrolyte	Applied potential	Urea yield rate	Faradic efficiency
Polypyrrole film^[^ ^15^ ^]^	30 bar CO_2_	30 bar N_2_	0.1 m Li_2_SO_4_ + 0.03 m H^+^	−0.325 V versus NHE	31.8 μg h^−1^ cm^−2^	6.9%
PdCu/TiO_2_‐400^[^ ^12^ ^]^	1 bar CO_2_	1 bar N_2_	1 m KHCO_3_	−0.4 V versus RHE	3.36 mmol h^−1^ g^−1^	8.92%
Bi/BiVO_4_^[^ ^18^ ^]^	1 bar CO_2_	1 bar N_2_	0.1 m KHCO_3_	−0.4 V versus RHE	5.91 mmol h^−1^ g^−1^	12.55%
BiFeO_3_/BiVO_4_^[^ ^19^ ^]^	1 bar CO_2_	1 bar N_2_	0.1 m KHCO_3_	−0.4 V versus RHE	4.94 mmol h^−1^ g^−1^	17.18%
Te–Pd NCs^[^ ^31^ ^]^	1 bar CO_2_	0.01 m NO_2_^−^	0.1 m KHCO_3_ + 0.01 m KNO_2_	−1.1 V versus RHE	−	12.2%
ZnO–V^[^ ^32^ ^]^	1 bar CO_2_	0.1 m NO_2_^−^	0.2 m NaHCO_3_ + 0.1 m NaNO_2_	−0.79 V versus RHE	5.52 mmol h^−1^ cm^−2^	23.26%
Cu–TiO_2_–V_o_^[^ ^33^ ^]^	1 bar CO_2_	0.02 m NO_2_^−^	0.2 m KHCO_3_ + 0.02 m KNO_2_	−0.4 V versus RHE	20.8 μmol h^−1^	43.1%
Cu^[^ ^26^ ^]^	1 bar CO_2_	0.02 m NO_2_^−^	0.2 m KHCO_3_ + 0.02 m KNO_2_	−0.75 V versus SHE	−	37%
Cd^[28a]^	1 bar CO_2_	0.02 m NO_2_^−^	0.2 m KHCO_3_ + 0.02 m KNO_2_	−1.0 V versus SHE	−	55%
ZrB_2_ ^[28b]^	1 bar CO_2_	0.02 m NO_2_^−^	0.2 KHCO_3_ + 0.02 m KNO_2_	−1.3 V vs SHE	−	33%
Ni–phthalocyanine^[28c]^	1 bar CO_2_	0.02 m NO_2_^−^	0.2 m KHCO_3_ + 0.02 m KNO_2_	−1.5 V versus SHE	−	40%
Zn^[^ ^29^ ^]^	1 bar CO_2_	0.02 m NO_3_^−^	0.2 m KHCO_3_ + 0.02 M KNO_3_	−1.75 V versus SHE	−	35%

**Figure 8:**
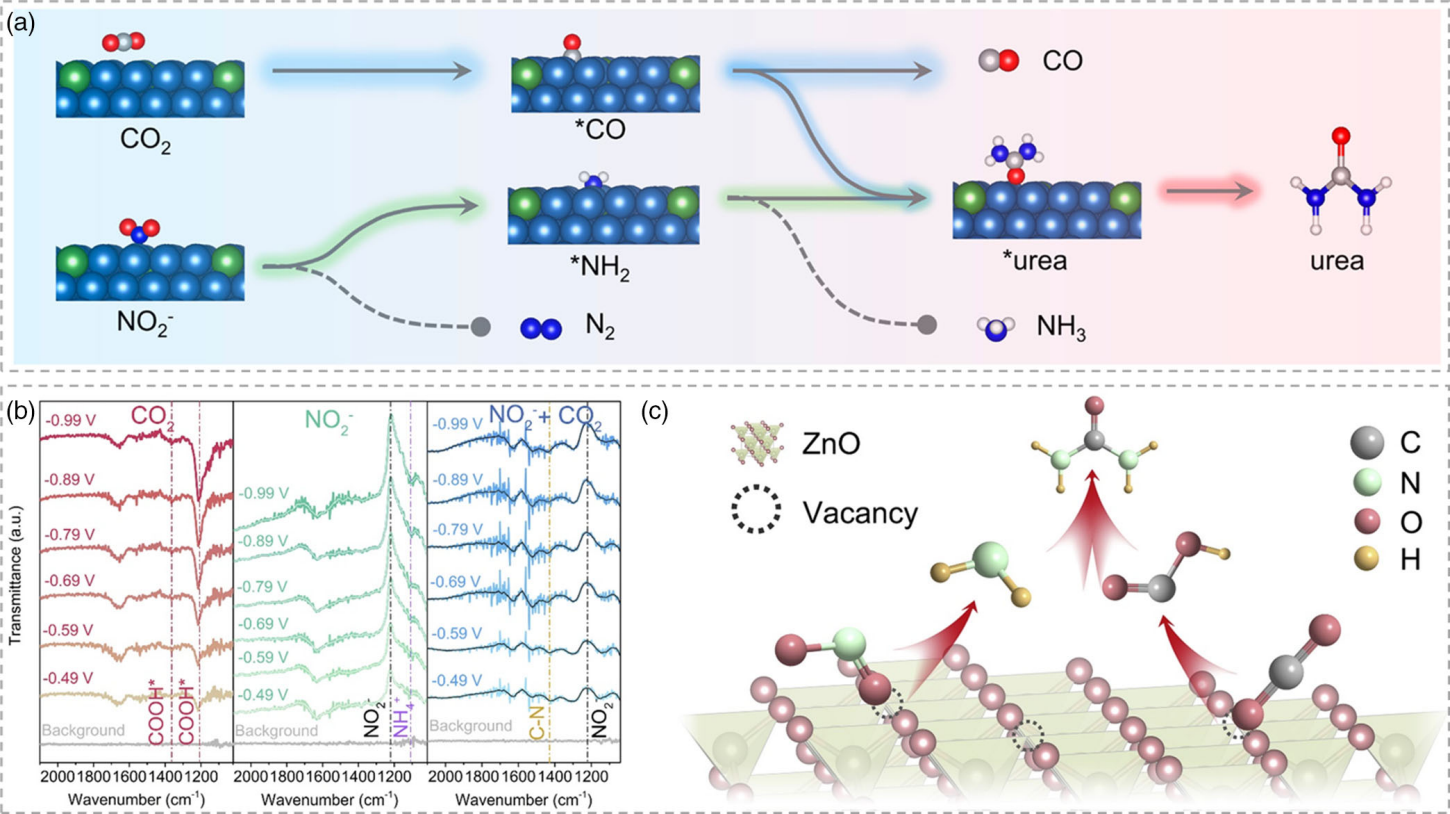
Reaction mechanism studies for electrocatalytic urea synthesis from carbon dioxide and nitrite ion coupling. a) Scheme of the urea synthesis on Te–Pd NCs. Reproduced with permission.^[^ ^31^ ^]^ Copyright 2020, American Chemical Society. b) In situ diffuse reflectance infrared Fourier transform spectroscopy spectra of ZnO–V under carbon dioxide, nitrite ions, and both. c) Schematic illustration of urea formation over ZnO–V. Reproduced with permission.^[^ ^32^ ^]^ Copyright 2021, Elsevier Ltd.

The mechanism of electrochemical urea synthesis from coupling of carbon dioxide and nitrite/nitrate ions has been studied. Most of the reports demonstrated that urea formation is derived from the reaction between CO* and NH_2_* intermediate species based on the comparative experimental results (**Figure** **8**a).^[^ ^31^, ^34^ ^]^ Replacing the feedstock from nitrite/nitrate to ammonia or carbon dioxide to carbon monoxide would not yield urea products. Feng et al. proved the feasibility of this mechanism by theoretical calculations.^[^ ^31^ ^]^ However, according to Meng's report, the urea formation pathway of COOH* and NH_2_* coupling has been proposed by means of online differential electrochemical mass spectrometry and in situ diffuse reflectance infrared Fourier transform spectroscopy (Figure 8b,c).^[^ ^32^ ^]^ The mechanism of urea synthesis is still controversial, and further research is needed to clarify the reaction pathways.

## 4 Conclusion and Outlook

The electrocatalytic coupling of C—N bond for urea synthesis is an energy‐saving and environment‐friendly alternative for the industrial urea synthetic process. With rational design of the electrocatalysts and optimization of the catalytic system, the effective utilization of resources and the expansion application range of electrocatalysis have been realized. However, the reaction mechanism of electrocatalytic urea synthesis is controversial at present, difficult to guide the design of the efficient catalyst theoretically. Meanwhile, the active site and the proposed intermediate attributed to urea formation are still uncertain. Thus, the existing problems of electrocoupling for urea synthesis and the direction of efforts are prospected. 1) For nitrogen and carbon dioxide coupling to produce urea, nitrogen molecules with high chemical inertness and nonpolar property are more difficult to adsorb and activate than carbon dioxide ones; thus, the catalytic activity of nitrogen should be given priority in catalyst design. The studies of nitrogen reduction reaction and electrocatalytic urea synthesis are complementary to each other. 2) The reaction mechanism of urea synthesis cannot be deduced retrospectively, only through comparative experiments. It is necessary to develop and apply advanced in situ characterization techniques to clarify the mechanism of urea synthesis reaction and identify electrocatalytic active sites, including the investigations of the adsorption and activation sites of each reactant, the site of coupling reaction, the intermediate interaction mechanism, and the driving force. 3) It is necessary to study and analyze the reaction pathways of the subreactions (carbon dioxide reduction, nitrogen reduction, and nitrite/nitrate reduction), the product selectivity, and efficiency on the electrocatalytic performance of urea synthesis, so as to guide the rational design of urea electrocatalysts. Meanwhile, the performance of urea synthesis should be improved from the perspective of catalytic system optimization, such as the application of ionic liquids as electrolyte with high gas solubility and the design of the reactor. 4) The demonstrated approaches in this review have realized the electrocatalytic coupling of C—N bond for urea synthesis, and other carbon sources such as some organic matter can also be used in the C–N coupling reaction to expand the application of electrocatalytic amine synthesis. It is necessary to regulate and balance the adsorption behavior of carbon species and nitrogen on the catalyst surface, and both the driving force for coupling and the identification of active sites are required to be investigated.

## Conflict of Interest

The authors declare no conflict of interest.
